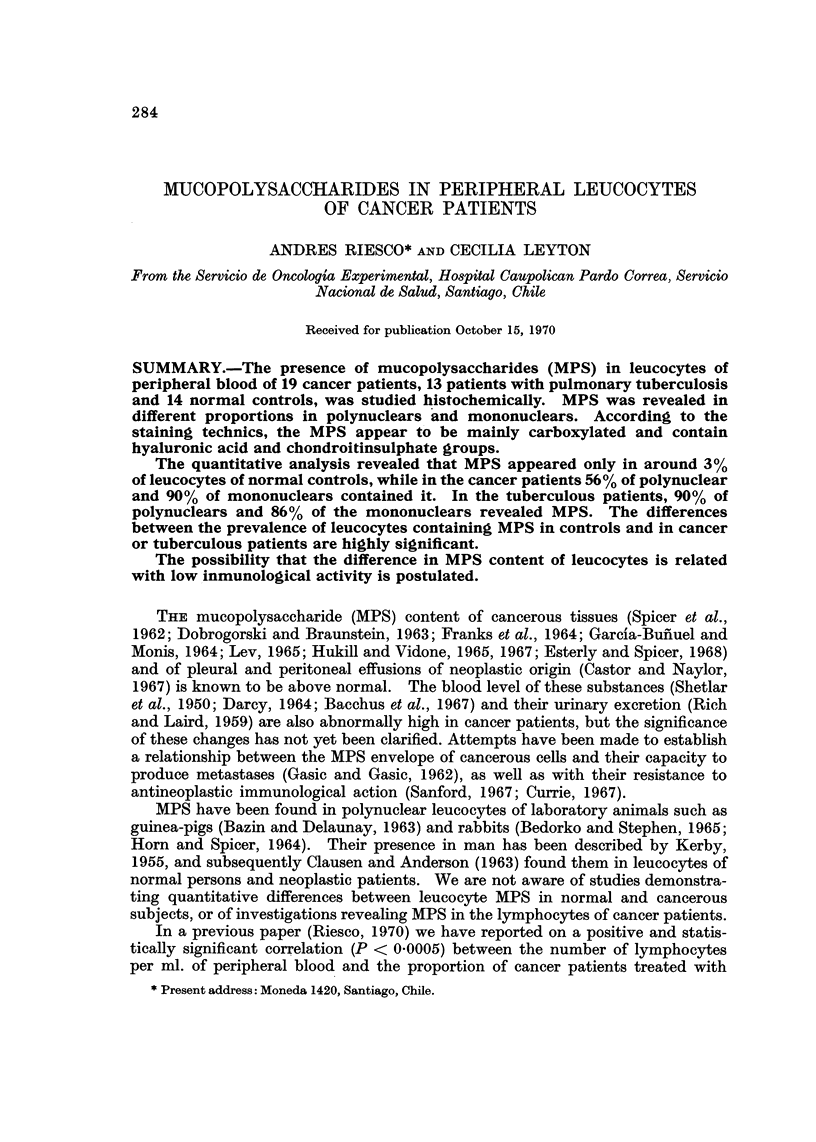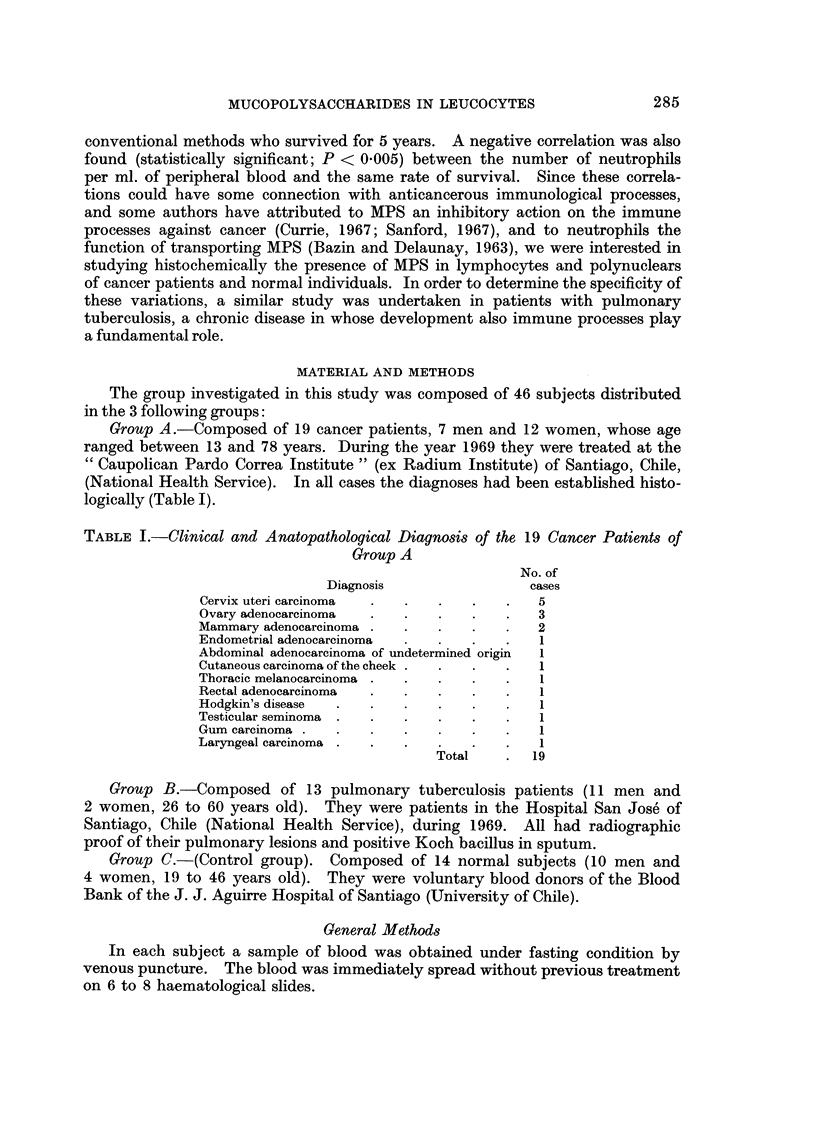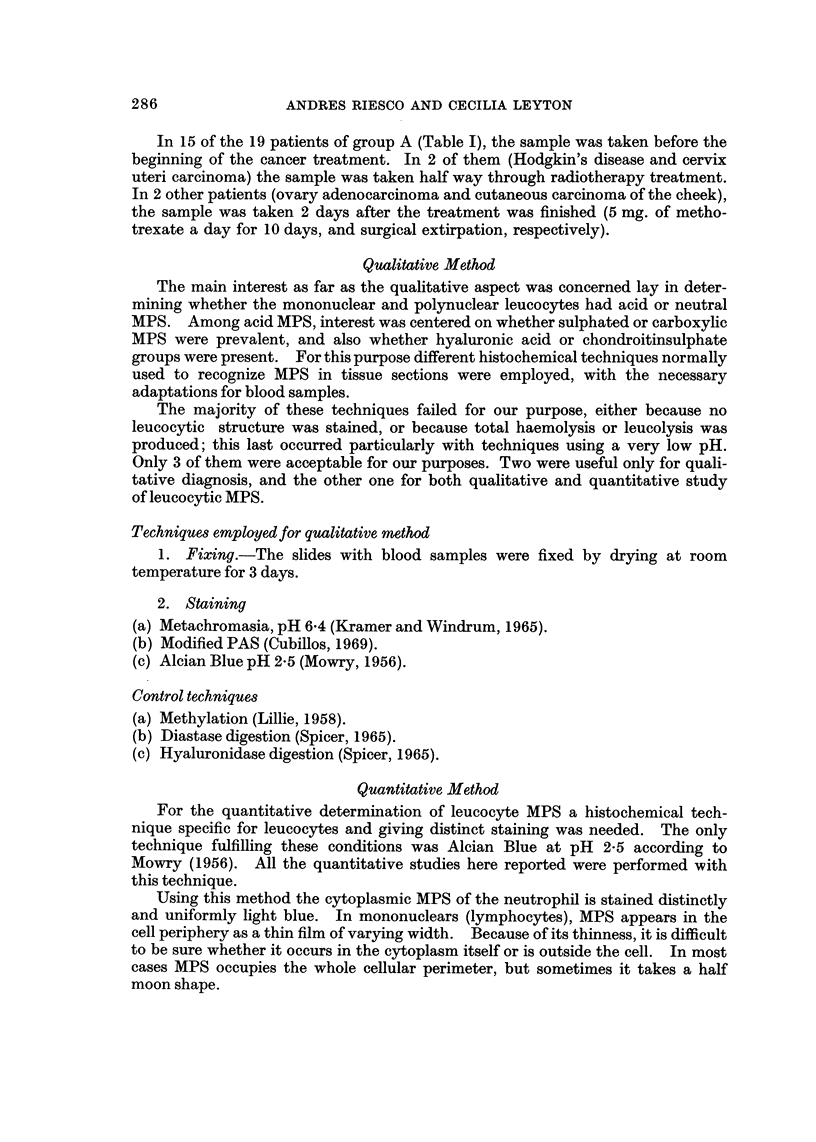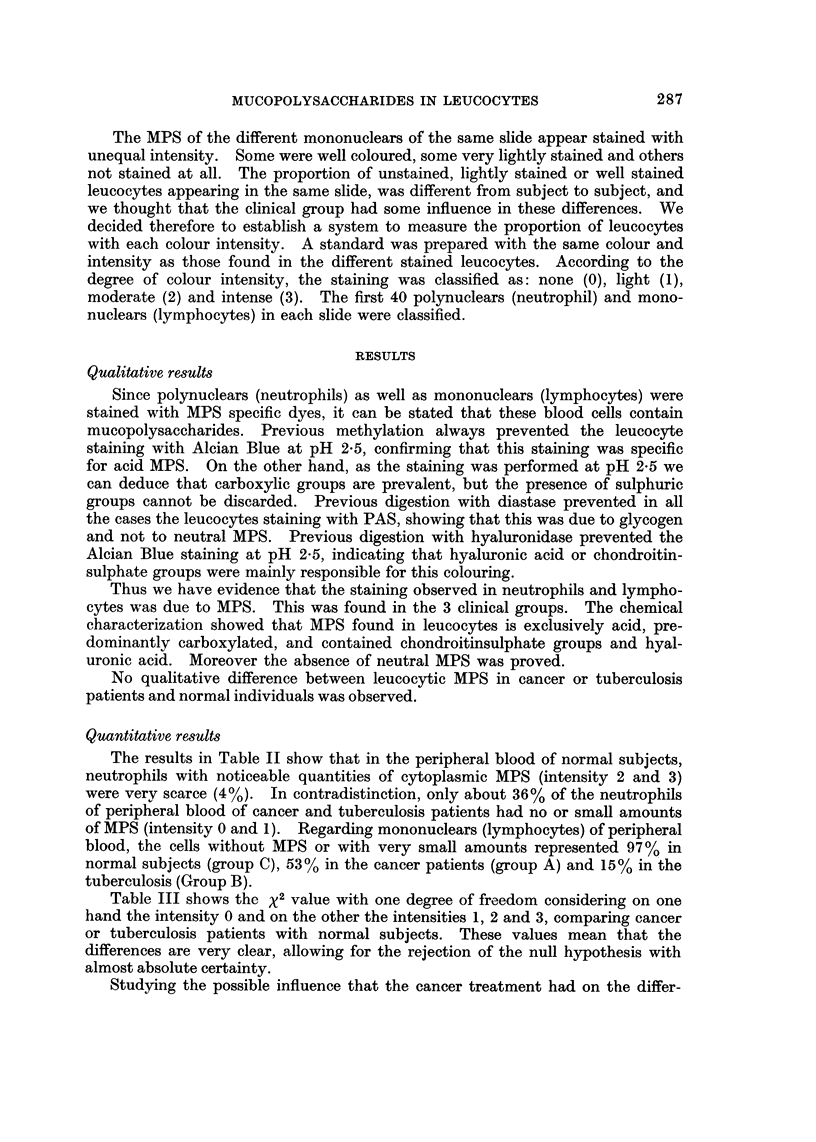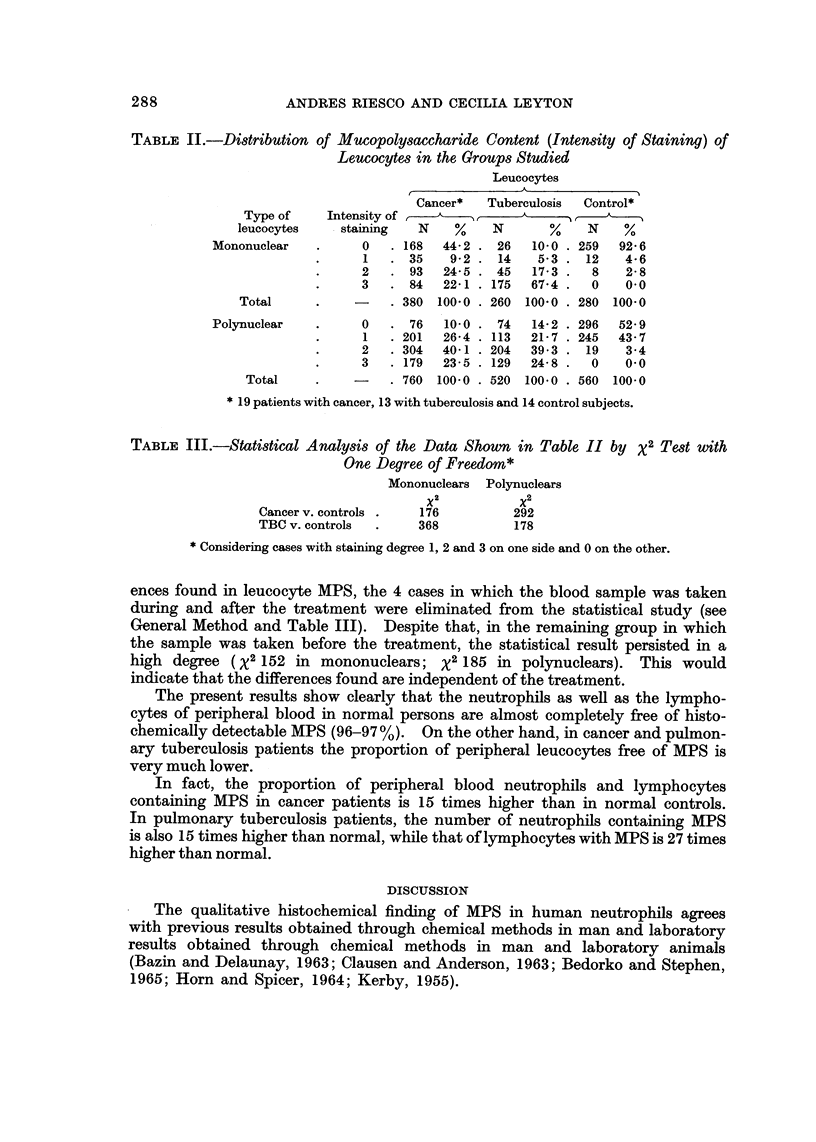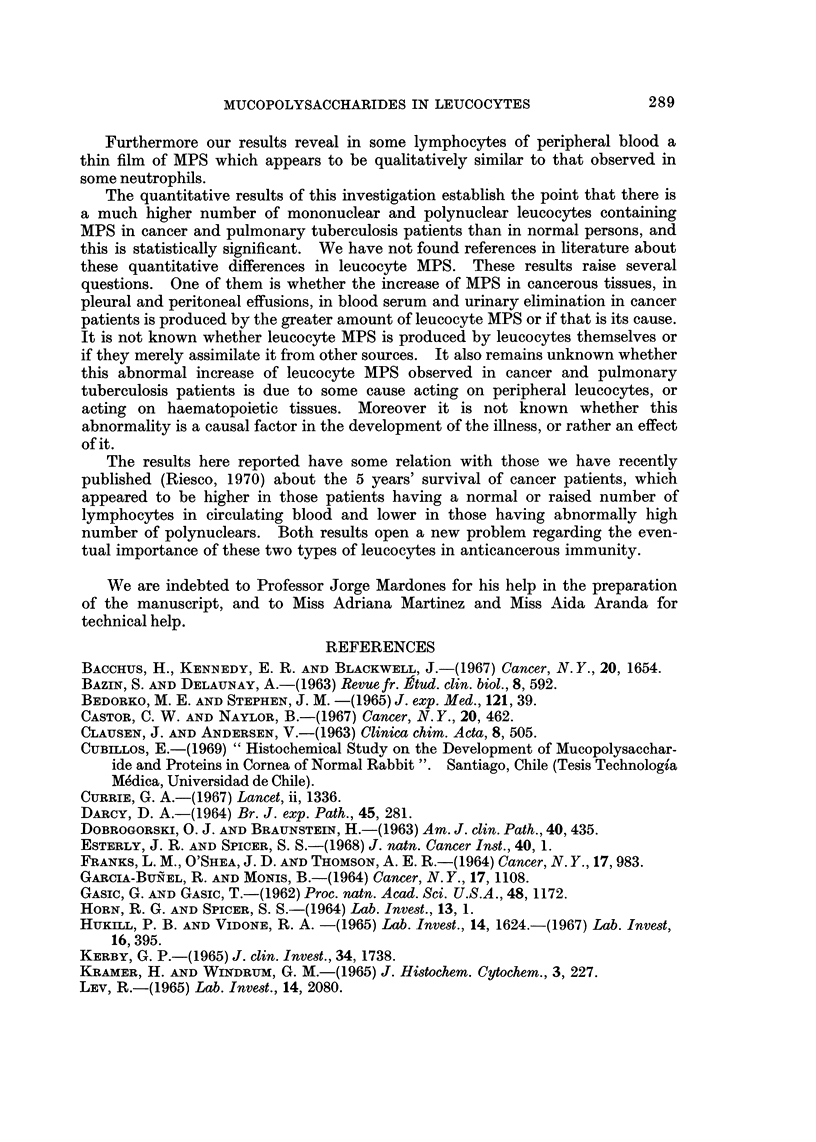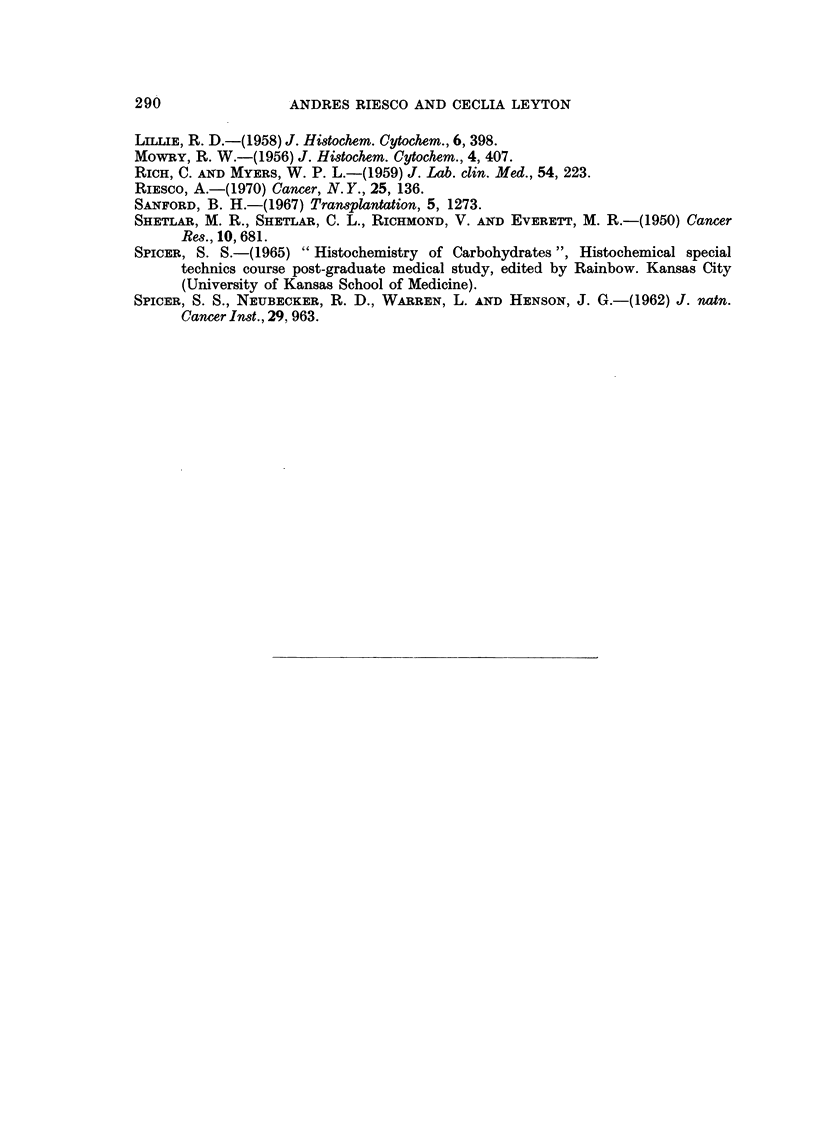# Mucopolysaccharides in Peripheral Leucocytes of Cancer Patients

**DOI:** 10.1038/bjc.1971.36

**Published:** 1971-06

**Authors:** Andres Riesco, Cecilia Leyton

## Abstract

The presence of mucopolysaccharides (MPS) in leucocytes of peripheral blood of 19 cancer patients, 13 patients with pulmonary tuberculosis and 14 normal controls, was studied histochemically. MPS was revealed in different proportions in polynuclears and mononuclears. According to the staining technics, the MPS appear to be mainly carboxylated and contain hyaluronic acid and chondroitinsulphate groups.

The quantitative analysis revealed that MPS appeared only in around 3% of leucocytes of normal controls, while in the cancer patients 56% of polynuclear and 90% of mononuclears contained it. In the tuberculous patients, 90% of polynuclears and 86% of the mononuclears revealed MPS. The differences between the prevalence of leucocytes containing MPS in controls and in cancer or tuberculous patients are highly significant.

The possibility that the difference in MPS content of leucocytes is related with low inmunological activity is postulated.


### MUCOPOLYSACCHARIDES IN PERIPHERAL LEUCOCYTES OF CANCER PATIENTS

ANDRES RIESCO* AND CECILIA LEYTON

*From the Servicio de Oncología Experimental, Hospital Caupolican Pardo Correa, Servicio Nacional de Salud, Santiago, Chile*

Received for publication October 15, 1970

**SUMMARY.—The presence of mucopolysaccharides (MPS) in leucocytes of peripheral blood of 19 cancer patients, 13 patients with pulmonary tuberculosis and 14 normal controls, was studied histochemically. MPS was revealed in different proportions in polynuclears and mononuclears. According to the staining technics, the MPS appear to be mainly carboxylated and contain hyaluronic acid and chondroitinsulphate groups.**

**The quantitative analysis revealed that MPS appeared only in around 3% of leucocytes of normal controls, while in the cancer patients 56% of polynuclear and 90% of mononuclears contained it. In the tuberculous patients, 90% of polynuclears and 86% of the mononuclears revealed MPS. The differences between the prevalence of leucocytes containing MPS in controls and in cancer or tuberculous patients are highly significant.**

**The possibility that the difference in MPS content of leucocytes is related with low inmunological activity is postulated.**

THE mucopolysaccharide (MPS) content of cancerous tissues (Spicer *et al.*, 1962; Dobrogorski and Braunstein, 1963; Franks *et al.*, 1964; García-Buñuel and Monis, 1964; Lev, 1965; Hukill and Vidone, 1965, 1967; Esterly and Spicer, 1968) and of pleural and peritoneal effusions of neoplastic origin (Castor and Naylor, 1967) is known to be above normal. The blood level of these substances (Shetlar *et al.*, 1950; Darcy, 1964; Bacchus *et al.*, 1967) and their urinary excretion (Rich and Laird, 1959) are also abnormally high in cancer patients, but the significance of these changes has not yet been clarified. Attempts have been made to establish a relationship between the MPS envelope of cancerous cells and their capacity to produce metastases (Gasic and Gasic, 1962), as well as with their resistance to antineoplastic immunological action (Sanford, 1967; Currie, 1967).

MPS have been found in polynuclear leucocytes of laboratory animals such as guinea-pigs (Bazin and Delaunay, 1963) and rabbits (Bedorko and Stephen, 1965; Horn and Spicer, 1964). Their presence in man has been described by Kerby, 1955, and subsequently Clausen and Anderson (1963) found them in leucocytes of normal persons and neoplastic patients. We are not aware of studies demonstrating quantitative differences between leucocyte MPS in normal and cancerous subjects, or of investigations revealing MPS in the lymphocytes of cancer patients.

In a previous paper (Riesco, 1970) we have reported on a positive and statistically significant correlation ($P < 0{\cdot}0005$) between the number of lymphocytes per ml. of peripheral blood and the proportion of cancer patients treated with

* Present address: Moneda 1420, Santiago, Chile.

conventional methods who survived for 5 years. A negative correlation was also found (statistically significant; $P < 0 \cdot 005$) between the number of neutrophils per ml. of peripheral blood and the same rate of survival. Since these correlations could have some connection with anticancerous immunological processes, and some authors have attributed to MPS an inhibitory action on the immune processes against cancer (Currie, 1967; Sanford, 1967), and to neutrophils the function of transporting MPS (Bazin and Delaunay, 1963), we were interested in studying histochemically the presence of MPS in lymphocytes and polynuclears of cancer patients and normal individuals. In order to determine the specificity of these variations, a similar study was undertaken in patients with pulmonary tuberculosis, a chronic disease in whose development also immune processes play a fundamental role.

### MATERIAL AND METHODS

The group investigated in this study was composed of 46 subjects distributed in the 3 following groups:

*Group A.*—Composed of 19 cancer patients, 7 men and 12 women, whose age ranged between 13 and 78 years. During the year 1969 they were treated at the " Caupolican Pardo Correa Institute " (ex Radium Institute) of Santiago, Chile, (National Health Service). In all cases the diagnoses had been established histologically (Table I).

TABLE I.—*Clinical and Anatopathological Diagnosis of the* 19 *Cancer Patients of Group A*

| Diagnosis | No. of cases |
|---|---|
| Cervix uteri carcinoma | 5 |
| Ovary adenocarcinoma | 3 |
| Mammary adenocarcinoma | 2 |
| Endometrial adenocarcinoma | 1 |
| Abdominal adenocarcinoma of undetermined origin | 1 |
| Cutaneous carcinoma of the cheek | 1 |
| Thoracic melanocarcinoma | 1 |
| Rectal adenocarcinoma | 1 |
| Hodgkin's disease | 1 |
| Testicular seminoma | 1 |
| Gum carcinoma | 1 |
| Laryngeal carcinoma | 1 |
| Total | 19 |

*Group B.*—Composed of 13 pulmonary tuberculosis patients (11 men and 2 women, 26 to 60 years old). They were patients in the Hospital San José of Santiago, Chile (National Health Service), during 1969. All had radiographic proof of their pulmonary lesions and positive Koch bacillus in sputum.

*Group C.*—(Control group). Composed of 14 normal subjects (10 men and 4 women, 19 to 46 years old). They were voluntary blood donors of the Blood Bank of the J. J. Aguirre Hospital of Santiago (University of Chile).

### *General Methods*

In each subject a sample of blood was obtained under fasting condition by venous puncture. The blood was immediately spread without previous treatment on 6 to 8 haematological slides.

In 15 of the 19 patients of group A (Table I), the sample was taken before the beginning of the cancer treatment. In 2 of them (Hodgkin's disease and cervix uteri carcinoma) the sample was taken half way through radiotherapy treatment. In 2 other patients (ovary adenocarcinoma and cutaneous carcinoma of the cheek), the sample was taken 2 days after the treatment was finished (5 mg. of methotrexate a day for 10 days, and surgical extirpation, respectively).

### *Qualitative Method*

The main interest as far as the qualitative aspect was concerned lay in determining whether the mononuclear and polynuclear leucocytes had acid or neutral MPS. Among acid MPS, interest was centered on whether sulphated or carboxylic MPS were prevalent, and also whether hyaluronic acid or chondroitinsulphate groups were present. For this purpose different histochemical techniques normally used to recognize MPS in tissue sections were employed, with the necessary adaptations for blood samples.

The majority of these techniques failed for our purpose, either because no leucocytic structure was stained, or because total haemolysis or leucolysis was produced; this last occurred particularly with techniques using a very low pH. Only 3 of them were acceptable for our purposes. Two were useful only for qualitative diagnosis, and the other one for both qualitative and quantitative study of leucocytic MPS.

#### *Techniques employed for qualitative method*

1. *Fixing.*—The slides with blood samples were fixed by drying at room temperature for 3 days.

2. *Staining*

(a) Metachromasia, pH 6·4 (Kramer and Windrum, 1965).
(b) Modified PAS (Cubillos, 1969).
(c) Alcian Blue pH 2·5 (Mowry, 1956).

#### *Control techniques*

(a) Methylation (Lillie, 1958).
(b) Diastase digestion (Spicer, 1965).
(c) Hyaluronidase digestion (Spicer, 1965).

### *Quantitative Method*

For the quantitative determination of leucocyte MPS a histochemical technique specific for leucocytes and giving distinct staining was needed. The only technique fulfilling these conditions was Alcian Blue at pH 2·5 according to Mowry (1956). All the quantitative studies here reported were performed with this technique.

Using this method the cytoplasmic MPS of the neutrophil is stained distinctly and uniformly light blue. In mononuclears (lymphocytes), MPS appears in the cell periphery as a thin film of varying width. Because of its thinness, it is difficult to be sure whether it occurs in the cytoplasm itself or is outside the cell. In most cases MPS occupies the whole cellular perimeter, but sometimes it takes a half moon shape.

The MPS of the different mononuclears of the same slide appear stained with unequal intensity. Some were well coloured, some very lightly stained and others not stained at all. The proportion of unstained, lightly stained or well stained leucocytes appearing in the same slide, was different from subject to subject, and we thought that the clinical group had some influence in these differences. We decided therefore to establish a system to measure the proportion of leucocytes with each colour intensity. A standard was prepared with the same colour and intensity as those found in the different stained leucocytes. According to the degree of colour intensity, the staining was classified as: none (0), light (1), moderate (2) and intense (3). The first 40 polynuclears (neutrophil) and mononuclears (lymphocytes) in each slide were classified.

### RESULTS

### *Qualitative results*

Since polynuclears (neutrophils) as well as mononuclears (lymphocytes) were stained with MPS specific dyes, it can be stated that these blood cells contain mucopolysaccharides. Previous methylation always prevented the leucocyte staining with Alcian Blue at pH 2·5, confirming that this staining was specific for acid MPS. On the other hand, as the staining was performed at pH 2·5 we can deduce that carboxylic groups are prevalent, but the presence of sulphuric groups cannot be discarded. Previous digestion with diastase prevented in all the cases the leucocytes staining with PAS, showing that this was due to glycogen and not to neutral MPS. Previous digestion with hyaluronidase prevented the Alcian Blue staining at pH 2·5, indicating that hyaluronic acid or chondroitinsulphate groups were mainly responsible for this colouring.

Thus we have evidence that the staining observed in neutrophils and lymphocytes was due to MPS. This was found in the 3 clinical groups. The chemical characterization showed that MPS found in leucocytes is exclusively acid, predominantly carboxylated, and contained chondroitinsulphate groups and hyaluronic acid. Moreover the absence of neutral MPS was proved.

No qualitative difference between leucocytic MPS in cancer or tuberculosis patients and normal individuals was observed.

### *Quantitative results*

The results in Table II show that in the peripheral blood of normal subjects, neutrophils with noticeable quantities of cytoplasmic MPS (intensity 2 and 3) were very scarce (4%). In contradistinction, only about 36% of the neutrophils of peripheral blood of cancer and tuberculosis patients had no or small amounts of MPS (intensity 0 and 1). Regarding mononuclears (lymphocytes) of peripheral blood, the cells without MPS or with very small amounts represented 97% in normal subjects (group C), 53% in the cancer patients (group A) and 15% in the tuberculosis (Group B).

Table III shows the $\chi^2$ value with one degree of freedom considering on one hand the intensity 0 and on the other the intensities 1, 2 and 3, comparing cancer or tuberculosis patients with normal subjects. These values mean that the differences are very clear, allowing for the rejection of the null hypothesis with almost absolute certainty.

Studying the possible influence that the cancer treatment had on the differ-

TABLE II.—*Distribution of Mucopolysaccharide Content (Intensity of Staining) of Leucocytes in the Groups Studied*

| Type of leucocytes | Intensity of staining | Leucocytes: Cancer* N | % | Tuberculosis N | % | Control* N | % |
|---|---|---|---|---|---|---|---|
| Mononuclear | 0 | 168 | 44·2 | 26 | 10·0 | 259 | 92·6 |
| | 1 | 35 | 9·2 | 14 | 5·3 | 12 | 4·6 |
| | 2 | 93 | 24·5 | 45 | 17·3 | 8 | 2·8 |
| | 3 | 84 | 22·1 | 175 | 67·4 | 0 | 0·0 |
| Total | — | 380 | 100·0 | 260 | 100·0 | 280 | 100·0 |
| Polynuclear | 0 | 76 | 10·0 | 74 | 14·2 | 296 | 52·9 |
| | 1 | 201 | 26·4 | 113 | 21·7 | 245 | 43·7 |
| | 2 | 304 | 40·1 | 204 | 39·3 | 19 | 3·4 |
| | 3 | 179 | 23·5 | 129 | 24·8 | 0 | 0·0 |
| Total | — | 760 | 100·0 | 520 | 100·0 | 560 | 100·0 |

* 19 patients with cancer, 13 with tuberculosis and 14 control subjects.

TABLE III.—*Statistical Analysis of the Data Shown in Table II by $\chi^2$ Test with One Degree of Freedom**

| | Mononuclears $\chi^2$ | Polynuclears $\chi^2$ |
|---|---|---|
| Cancer v. controls | 176 | 292 |
| TBC v. controls | 368 | 178 |

* Considering cases with staining degree 1, 2 and 3 on one side and 0 on the other.

ences found in leucocyte MPS, the 4 cases in which the blood sample was taken during and after the treatment were eliminated from the statistical study (see General Method and Table III). Despite that, in the remaining group in which the sample was taken before the treatment, the statistical result persisted in a high degree ($\chi^2$ 152 in mononuclears; $\chi^2$ 185 in polynuclears). This would indicate that the differences found are independent of the treatment.

The present results show clearly that the neutrophils as well as the lymphocytes of peripheral blood in normal persons are almost completely free of histochemically detectable MPS (96–97%). On the other hand, in cancer and pulmonary tuberculosis patients the proportion of peripheral leucocytes free of MPS is very much lower.

In fact, the proportion of peripheral blood neutrophils and lymphocytes containing MPS in cancer patients is 15 times higher than in normal controls. In pulmonary tuberculosis patients, the number of neutrophils containing MPS is also 15 times higher than normal, while that of lymphocytes with MPS is 27 times higher than normal.

### DISCUSSION

The qualitative histochemical finding of MPS in human neutrophils agrees with previous results obtained through chemical methods in man and laboratory results obtained through chemical methods in man and laboratory animals (Bazin and Delaunay, 1963; Clausen and Anderson, 1963; Bedorko and Stephen, 1965; Horn and Spicer, 1964; Kerby, 1955).

Furthermore our results reveal in some lymphocytes of peripheral blood a thin film of MPS which appears to be qualitatively similar to that observed in some neutrophils.

The quantitative results of this investigation establish the point that there is a much higher number of mononuclear and polynuclear leucocytes containing MPS in cancer and pulmonary tuberculosis patients than in normal persons, and this is statistically significant. We have not found references in literature about these quantitative differences in leucocyte MPS. These results raise several questions. One of them is whether the increase of MPS in cancerous tissues, in pleural and peritoneal effusions, in blood serum and urinary elimination in cancer patients is produced by the greater amount of leucocyte MPS or if that is its cause. It is not known whether leucocyte MPS is produced by leucocytes themselves or if they merely assimilate it from other sources. It also remains unknown whether this abnormal increase of leucocyte MPS observed in cancer and pulmonary tuberculosis patients is due to some cause acting on peripheral leucocytes, or acting on haematopoietic tissues. Moreover it is not known whether this abnormality is a causal factor in the development of the illness, or rather an effect of it.

The results here reported have some relation with those we have recently published (Riesco, 1970) about the 5 years' survival of cancer patients, which appeared to be higher in those patients having a normal or raised number of lymphocytes in circulating blood and lower in those having abnormally high number of polynuclears. Both results open a new problem regarding the eventual importance of these two types of leucocytes in anticancerous immunity.

We are indebted to Professor Jorge Mardones for his help in the preparation of the manuscript, and to Miss Adriana Martinez and Miss Aida Aranda for technical help.

### REFERENCES

BACCHUS, H., KENNEDY, E. R. AND BLACKWELL, J.—(1967) *Cancer, N.Y.*, **20**, 1654.
BAZIN, S. AND DELAUNAY, A.—(1963) *Revue fr. Étud. clin. biol.*, **8**, 592.
BEDORKO, M. E. AND STEPHEN, J. M. —(1965) *J. exp. Med.*, **121**, 39.
CASTOR, C. W. AND NAYLOR, B.—(1967) *Cancer, N.Y.*, **20**, 462.
CLAUSEN, J. AND ANDERSEN, V.—(1963) *Clinica chim. Acta*, **8**, 505.
CUBILLOS, E.—(1969) " Histochemical Study on the Development of Mucopolysaccharide and Proteins in Cornea of Normal Rabbit ". Santiago, Chile (Tesis Technología Médica, Universidad de Chile).
CURRIE, G. A.—(1967) *Lancet*, ii, 1336.
DARCY, D. A.—(1964) *Br. J. exp. Path.*, **45**, 281.
DOBROGORSKI, O. J. AND BRAUNSTEIN, H.—(1963) *Am. J. clin. Path.*, **40**, 435.
ESTERLY, J. R. AND SPICER, S. S.—(1968) *J. natn. Cancer Inst.*, **40**, 1.
FRANKS, L. M., O'SHEA, J. D. AND THOMSON, A. E. R.—(1964) *Cancer, N.Y.*, **17**, 983.
GARCIA-BUÑEL, R. AND MONIS, B.—(1964) *Cancer, N.Y.*, **17**, 1108.
GASIC, G. AND GASIC, T.—(1962) *Proc. natn. Acad. Sci. U.S.A.*, **48**, 1172.
HORN, R. G. AND SPICER, S. S.—(1964) *Lab. Invest.*, **13**, 1.
HUKILL, P. B. AND VIDONE, R. A. —(1965) *Lab. Invest.*, **14**, 1624.—(1967) *Lab. Invest*, **16**, 395.
KERBY, G. P.—(1965) *J. clin. Invest.*, **34**, 1738.
KRAMER, H. AND WINDRUM, G. M.—(1965) *J. Histochem. Cytochem.*, **3**, 227.
LEV, R.—(1965) *Lab. Invest.*, **14**, 2080.

LILLIE, R. D.—(1958) *J. Histochem. Cytochem.*, **6**, 398.
MOWRY, R. W.—(1956) *J. Histochem. Cytochem.*, **4**, 407.
RICH, C. AND MYERS, W. P. L.—(1959) *J. Lab. clin. Med.*, **54**, 223.
RIESCO, A.—(1970) *Cancer, N.Y.*, **25**, 136.
SANFORD, B. H.—(1967) *Transplantation*, **5**, 1273.
SHETLAR, M. R., SHETLAR, C. L., RICHMOND, V. AND EVERETT, M. R.—(1950) *Cancer Res.*, **10**, 681.
SPICER, S. S.—(1965) " Histochemistry of Carbohydrates ", Histochemical special technics course post-graduate medical study, edited by Rainbow. Kansas City (University of Kansas School of Medicine).
SPICER, S. S., NEUBECKER, R. D., WARREN, L. AND HENSON, J. G.—(1962) *J. natn. Cancer Inst.*, **29**, 963.